# Importance of Mineral Elements of Food

**Published:** 1881-11

**Authors:** 


### IMPORTANCE OF MINERAL ELEMENTS OF FOOD.

"The more a man follows nature," says Hufeland, in his Art of Prolonging Life, "and is obedient to her laws, the longer he will live; the further he deviates from these, the shorter will be his existence." Nature has ready prepared abundance of food for man, and if he partakes of it as he has need, in nearly the form offered, there is no danger of getting far out of the way. The art of man, however, steps in, and changes, takes from and refines many articles of food, so that their original constitution is materially changed. How far such changes modify the healthfulness of food is a proper subject for inquiry.

Wheat, as furnished by nature, is a wholesome and most valuable food. In early times it was boiled until soft, or ground and made into bread, furnishing people with healthful food. In later times, to gratify the taste and please the eye, it has been customary to separate all of the bran portion of the ground wheat, leaving only the white and fine particles for the making of bread. One hundred pounds of wheat, when ground, will make about 76 pounds of flour and 20 pounds of bran. The flour contains of albuminoids as tissue-making elements, 1.65 per cent. and of phosphates and other salts 0.70 per cent., making a total of 2.35 per cent. The bran contains of albuminoids or tissue-making elements, 3.10, and of phosphates and other salts 7.05 per cent., making a total of 10.15 per cent. In other words, the bran, for the purposes of nutrition, is four-fold richer than the flour, the 20 pounds of bran made from 100 pounds of wheat containing more nutriment than the 76 pounds of flour. The flour consists chiefly of starch, while the flesh-forming elements, together with the blood and bone-producing constituents, are pricipally rejected in the bran. It will be observed that nearly all the phosphates and other salts are removed from the flour in grinding, leaving only 0.70 per cent., while the bran contains 7.05 per cent. of these important elements.

Is it probable that so large a proportion of the mineral elements of wheat can be thus rejected without ill effects to those who live largely upon a diet of flour bread? This is an important question for consideration; complete data for the decision of the question are not at present available, but some important facts have been established.

Numerous experiments in feeding farm stock have shown that, when animals are fed on food from which the phosphates and other salts have been removed as completely as possible, they become sleepy and weak, especially in the extremities, and finally die from lack of mineral food, although the quantity of organic food eaten and digested may be amply sufficient to sustain life. The digestion of food did not seem to be interfered with, and when the animals were examined after death, all the organs appeared healthy and well nourished. Now, if such marked and fatal results attend the removal of the phosphates and other salts from the food of domestic animals, is it not probable that the removal of nine-tenths of these same elements from wheat, in the process of the manufacture of flour, would be attended with injurious effects? Especially would this seem probable where children who, while growing, need large supplies of flesh, blood and bone material, are fed largely upon flour bread. Is it not probable that many of the pale, poorly nourished children, with small, flabby muscles, owe something of their condition to their diet of flour bread? Not all of the fat and plump children are really well nourished, as is shown by the smallness of their muscle development, and their general inability to withstand fatigue or diseases. Fat is not muscle, and does not impart strength.

Is it not a significant fact that there should be comparatively so little disease among our domestic animals—so rare that one dies, so rare that the young fail to grow up—while there is so much sickness among human beings and so large a proportion die before they are five years old? When any of the domestic animals are sick we attribute their sickness to improper food, or wrong management of some kind, and seek to remedy it. If one of them dies we do not say it is a "wise dispensation of Providence." We generally believe that there has been carelessness or mismanagement somewhere. As regards the human family we seem to take it for granted that there is to be a large amount of sickness and do not trouble ourselves very much to ascertain the cause of it. Inasmuch as the natural period of the life of man is longer than that of the domestic animals it would reasonably be expected that the young of the human family would be endowed with a greater degree of vitality and be less likely to die young. The fact that one child in every five of those born dies before it is a year old, and that one-third of all the children die before they are five years old, is full of significance and worthy of consideration. If one-fifth of all the calves born should die of disease before they become a year old we should have a government commission appointed to investigate the causes of the mortality; but the children continue to die and no one thinks of having the causes investigated.

It may be suggested that the eating of flour bread can have nothing to do with the death of children under one year of age, as they do not indulge much in that kind of food. True, they do not, or should not eat bread of any kind, but their mothers do, and a mother who lives largely on flour bread does not obtain enough bone and blood-making material to form a healthy body for her child, and her milk is nearly as poor in mineral elements as the flour bread which she eats. Thus, indirectly, even the infant may suffer from the removal of the phosphates from the wheat in grinding it.

Disease in plants is found to be associated sometimes with a deficiency of mineral elements of food in the soil. The potato rot has been ascribed, by good authority, to a deficiency of lime and magnesia in the soil. The amount of magnesia in the ash of sound potatoes is from 5 to 10 per cent., while in diseased tubers it was found to be less than 4 per cent. Of lime, sound potatoes contained 5 per cent., while diseased ones contained only 1.77 per cent. Diseased orange trees were found by Professor Thorpe to contain a deficiency of lime and magnesia. If a deficiency of mineral elements is associated with disease of plants, it is highly probable that a deficiency of necessary mineral elements may be a cause of disease in the human system. If the removal of the phosphates and other mineral elements from the food of domestic animals causes them to die, certainly it is at least probable that the removal of so large a proportion of the mineral elements from wheat, in the process of grinding, may exert a deleterious effect upon the health of those subsisting largely upon it. It is a subject worthy of further attention.—*New England Farmer.*

---

THE CURSE OF ALCOHOL.—Canon Farrar says: "He alone, by whom the hairs of our head are all numbered, can count the widows because of alcohol; the gray heads that it has made gray; the sad hearts that it has crushed with sadness; the brilliant minds which it has quenched; the unfolding promise which it has cankered; the bright and happy boys and girls whom it has blasted into misery; the young and gifted whom it has hurried along into dishonored and nameless graves."

